# A comprehensive joint replacement program for total knee arthroplasty: a descriptive study

**DOI:** 10.1186/1471-2474-9-154

**Published:** 2008-11-19

**Authors:** Jon R Cook, Meghan Warren, Kathleen J Ganley, Paul Prefontaine, Jack W Wylie

**Affiliations:** 1 Department of Rehabilitation Sciences, Verde Valley Medical Center, 269 S. Candy Lane, Cottonwood, AZ, USA; 2 Department of Physical Therapy and Athletic Training, Northern Arizona University, PO Box 15105, Flagstaff, AZ, USA; 3 Verde Valley Orthopedics, 450 S. Willard Street, Suite 101, Cottonwood, AZ, USA

## Abstract

**Background:**

Total knee arthroplasty (TKA) is a commonly performed surgical procedure in the US. It is important to have a comprehensive inpatient TKA program which maximizes outcomes while minimizing adverse events. The purpose of this study was to describe a TKA program – the Joint Replacement Program (JRP) – and report post-surgical outcomes.

**Methods:**

74 candidates for a primary TKA were enrolled in the JRP. The JRP was designed to minimize complications and optimize patient-centered outcomes using a team approach including the patient, patient's family, and a multidisciplinary team of health professionals. The JRP consisted of a pre-operative class, standard pathways for medical care, comprehensive peri-operative pain management, aggressive physical therapy (PT), and proactive discharge planning. Measures included functional tests, knee range of motion (ROM), and medical record abstraction of patient demographics, length of stay, discharge disposition, and complications over a 6-month follow-up period.

**Results:**

All patients achieved medical criteria for hospital discharge. The patients achieved the knee flexion ROM goal of 90° (91.7 ± 5.4°), but did not achieve the knee extension ROM goal of 0° (2.4 ± 2.6°). The length of hospital stay was two days for 53% of the patients, with 39% and 7% discharged in three and four days, respectively. All but three patients were discharged home with functional independence. 68% of these received outpatient physical therapy compared with 32% who received home physical therapy immediately after discharge. Two patients (< 3%) had medical complications during the inpatient hospital stay, and 9 patients (12%) had complications during the 6-month follow-up period.

**Conclusion:**

The comprehensive JRP for TKA was associated with satisfactory clinical outcomes, short lengths of stay, a high percentage of patients discharged home with outpatient PT, and minimal complications. This JRP may represent an efficient, effective and safe protocol for providing care after a TKA.

## Background

Total knee arthroplasty (TKA) is a common and successful surgical intervention for the management of disability secondary to osteoarthritis of the knee.[1] TKAs are associated with low peri-operative morbidity and improved pain and functional status.[2,3] Over 400,000 TKAs were performed in the United States in 2005.[4] This number is expected to increase dramatically over the coming decades secondary to the success of the intervention and the aging "baby boomer" population.[4,5]

The costs associated with TKAs (e.g. hospitalization, rehabilitation, etc.) are high. For example, Medicare reimburses over $2 billion each year for primary TKAs.[6,7] Medicare reimbursement rates are often much lower than those billed by hospitals. In the 2006 fiscal year, the national average charge for TKAs (and total hip arthroplasties) was $38,447, yet the national average reimbursement was $11,916.[8] Because of this discrepancy, some hospitals are electing to eliminate total joint replacement surgeries from their list of provided services. For others, the need to efficiently utilize healthcare resources while optimizing patient outcomes when caring for patients after TKAs is obvious.[4] To accomplish this, an emphasis is placed on reducing lengths of hospital stays and minimizing peri-operative complications (e.g. hypoxia, infection, pneumonia, thrombosis, etc.) as means of managing the costs associated with TKAs. [9-11] Each of the following has a potential to minimize length of stay and/or post-operative complications in some manner: pre-operative education, [12-14] peri- and post-operative pain management, [15-17] clinical pathways, [18-20] early and aggressive rehabilitation including physical therapy (PT),[10,21-24] and proactive discharge planning.[13,25]

To our knowledge, there is no literature showing the effect(s) of a comprehensive program which incorporates all of these components. Hence, we developed an evidence-based, comprehensive program for the management of TKA and implemented it at a regional medical center. The purpose of this prospective study was to describe the joint replacement program (JRP) for TKA's and report post-surgical outcomes over 6 months of follow-up.

## Methods

### Study design and sample

The data for this study come from the JRP at Verde Valley Medical Center, a 99-bed regional medical center in the rural Southwest. 87 consecutive patients were candidates for and underwent a primary TKA between April 2006 and November 2007. Of these, 85% (n = 74) were enrolled in the JRP based on the following inclusion and exclusion criteria. Criteria for inclusion included failed conservative management leading to painful and/or function-limiting osteoarthritis of the knee, clearance for surgery by primary care and/or medical specialists if indicated, the ability to participate in PT in a group environment during the hospital stay, and caregiver support at home. Exclusion criteria included chronic neurologic conditions such as post-stroke hemiparesis, Parkinson's disease, dementia, or any other condition which would preclude participation in group PT during the inpatient hospital stay. The primary reasons patients were not enrolled in the JRP were dementia or insufficient support at home. The nature, purpose and potential risks of the interventions were explained to each participant and written informed consent was obtained in accordance with procedures approved by the Institutional Review Board of Northern Arizona Healthcare.

### Joint Replacement Program

The JRP is a wellness-based program designed to optimize patient-centered outcomes and minimize complications for joint replacement. It was developed based on current evidence and expert opinion. Specifically, the JRP was modeled after published literature in pain management,[13,16,21,23,25] complication prevention,[23,26] and cost containment after TKA.[25] Furthermore, the JRP was refined after visits to three "Centers for Excellence" in joint replacement throughout the country.

The JRP involves a multidisciplinary, coordinated team that includes the patient and patient's family, orthopedist, anesthesiologist, nurses, physical and occupational therapists, and case manager. The JRP consists of a pre-operative patient education and planning class, comprehensive peri- and post-operative pain management, standard pathways for medical care, aggressive rehabilitation/PT, and proactive discharge planning. The JRP includes clinical goals, including a two to three day hospital stay and discharge home with outpatient physical therapy.

### Pre-Operative Care

Although published literature regarding the efficacy of pre-operative education is not conclusive, [12-14] a Cochrane Review concluded a positive association between pre-operative education and patient's anxiety.[14] The purpose of pre-operative education in the JRP was to lessen patients' anxiety by making them aware of post-operative rehabilitation and involving them in goal setting and discharge planning.

Patients attended a pre-operative educational session approximately 1–2 weeks prior to the scheduled surgery date. During this 4-hour class, patients were educated to the philosophy of the JRP, including aggressive, early rehabilitation to prevent complications and facilitate home discharge. The surgical procedure was described in detail with an emphasis on peri- and post-operative pain management. Patients were evaluated by anesthesiology for possible methods of sedation during surgery, and all necessary lab work and medical tests were completed. A physical therapist instructed the patients in the post-operative (hospital and home) exercise program (seated ankle pumps, assisted knee extension, assisted hamstring and calf stretches, and assisted heel slides) and PT goals for discharge from the hospital (Table 1). Additionally, performance (6 minute walk test [6MWT])[27] and self-administered (Knee Injury and Osteoarthritis Outcome Score [KOOS])[28] outcome measures were administered to establish a baseline to assess progress objectively.[29] A case manager discussed discharge planning, including expected length of hospital stay, post-discharge rehabilitation and medical care, potential adaptive equipment, home safety, and other discharge dispositions if discharge home was not possible. A tour of the orthopedic ward including the physical therapy room, patient rooms, and corridors was part of the session to allow the patients to become familiar with the post-operative environment. The patients were issued a notebook that contained a summary of relevant JRP information covered in the pre-operative visit.

**Table 1:** Physical therapy goals for discharge home

Active-assisted knee range of motion 0–90° in a seated position
Independent with transfers (supine to sit to stand from various surfaces [bed, chair, car]) either alone or with assistance of a caregiver

Independent ambulation for 150 feet with a wheeled walker

Independent with home exercise program using written instructions

Independent with stairs, if necessary for home environment, using an assistive device and/or caregiver assistance

### Surgical Procedure and Intra-operative Care

Pre-operative medications included a long-acting oral opioid.[30] Patients were also given a Cox II non-steroidal anti-inflammatory,[30,31] acetaminophen, anti-anxiety, and anti-emetic medications pre-operatively. Regional anesthesia has been associated with less intra-operative blood loss, lower incidence of DVT, and better post-operative pain management compared to general anesthesia.[31,32] We hypothesized that fewer complications and better pain control would allow early mobilization. Therefore, a short-acting spinal anesthesia was used with all patients in the JRP.

The same orthopedist performed all TKAs reported herein using a standard para-medial approach with cruciate-sparing prostheses (PFC Sigma Knee System, DePuy Orthopaedics, Inc., Warsaw, IN);[12,33,34] care was taken to minimize the size of the incision. After components were cemented, an intra-operative, 100 cc intra-capsular injection was given for local pain control and consisted of bipuvicaine, epinephrine, soluble morphine sulphate, and normal saline. After the tourniquet was deflated, bleeding was minimized using electrocautery, and the incision was closed without a drain. Patients received one unit of autologous blood intra-operatively to minimize syncopal episodes.

### Post-operative Care

#### Medical management

The prevention of complications while facilitating an early and safe discharge was a primary goal of the JRP. Patients were assessed daily by the treating orthopedist to evaluate progress and the risk for complications. Nursing care was delivered by a coordinated team of registered nurses and patient-care technicians with additional training in the JRP procedures. A clinical pathway was followed that included the pain management described above in addition to routine nursing care. Additionally, nurses encouraged patient independence with mobility and self care as indicated by the patient's abilities.

Patient-controlled analgesia (PCA) was individualized for appropriate dose and complemented other pain management for the immediate post-operative period.[35] The PCA was discontinued on post-operative day (POD) #1, and pain was managed with oral medications as needed. The timing of oral pain medications was coordinated with PT appointments. Cryotherapy was initiated in the immediate post-operative period and was continued as needed for management of pain and swelling.[36]

Venous thromboprophylaxis included both mechanical and pharmacological management.[26] Mechanical treatment consisted of intermittent calf pump devices (SCD Express™, Tyco Healthcare/Kendall, Mansfield, MA). Intermittent calf pumps were initiated on the non-operative leg pre-operatively and on the operative leg immediately after post-operative dressings were placed, and were maintained bilaterally throughout the hospital stay when the patient was in bed. Anti-embolism stockings (T.E.D.™, Tyco Healthcare/Kendall, Mansfield, MA) were worn bilaterally for 6 weeks after surgery. Other mechanical management consisted of ambulation and range of motion (ROM) exercises on the day of surgery (POD #0), and hourly calf pumps. Pharmacological management consisted of a loading dose of Warfarin for anti-coagulation initiated in the recovery room and continued for a 14-day period. Prothrombin time and an international normalized ratio (INR) were completed at regular intervals during the 14-day period of anti-coagulation to maintain INR between 1.5 and 2.0.

#### Early, Aggressive Rehabilitation

There is consistent support in the literature for early and aggressive physical therapy, or mobilization, following TKA.[10,21-25] Therefore, one of the cornerstones of the JRP was early and aggressive PT to maximize functional independence and achieve previously stated goals (Table 1). Physical therapy was typically initiated 2–4 hours after surgery. Vital signs and clinical signs and symptoms guided PT treatment and progression throughout the inpatient hospital stay. Physical therapy treatment on POD #0 optimally included an evaluation (review of physician's orders, past medical history, and patient's status), bed mobility and therapeutic exercise, transfers and full weight-bearing ambulation with an appropriate assistive device. Beginning on POD #1, patients were seen for PT twice per day in a group environment with other JRP patients. The patients completed activities of daily living, including dressing in community versus hospital attire, with the assistance of an occupational therapist or nurse prior to group exercise sessions. Active-assisted knee ROM was measured in the seated position after the exercise sessions. Family and friends were encouraged to attend PT sessions to participate and learn the home ROM program. Physical therapy included ambulation, and transfer and stair training on an individual basis. Progress toward PT discharge goals (Table 1) was monitored on a dry erase board in the patient's room.

#### Discharge planning

The JRP was designed to facilitate discharge to home, with outpatient PT for continued rehabilitation. The orthopedist, with input from the multidisciplinary team, determined readiness for discharge based on the following criteria: medical stability (i.e. no anticipated conditions that would require re-admission to the hospital), wound stability (i.e. no erythema, discharge or redness), INR between 1.5 and 2.0, pain controlled with oral medications, and progress towards PT goals. Patients who were unable to be discharged home with outpatient physical therapy because of sub-acute medical needs, unsafe mobility, and/or transportation issues were referred for appropriate post-acute care (e.g. skilled nursing or home health care). Proactive discharge planning by a registered nurse case manager, which was initiated during the pre-operative visit, was continued after surgery. Discharge planning assured the patient had all necessary adaptive equipment and arrangements for follow-up care. Follow-up in the orthopedist's office was planned for 2–3 weeks, 6 weeks, 3 months, 6 months, and yearly after surgery. Follow-up visits with the orthopedist included wound assessment, functional range of motion and radiograph examinations.

### Measurements

Measurements were completed during the pre-operative educational session as well as during the inpatient hospital stay. Pre-operatively, height and weight were measured using a wall-mounted stadiometer and digital scale (Taylor Precision Products, Oak Brook IL), and body mass index (BMI) was calculated by weight in kilograms divided by height in meters squared. During the pre-operative educational session, the 6MWT[27] and the KOOS[28] were administered. The 6MWT test was conducted in an 18 meter uncarpeted hallway, and patients were instructed to cover as much distance as possible within six minutes. Standardized encouragement was given at regular intervals. Patients were permitted to use an assistive device and rest during the testing time. The area was marked in meters and the distance traveled by each patient was measured at the end of six minutes. The reliability of the 6MWT was found to be acceptable in 17 patients scheduled for a total hip or knee arthroplasty (correlation coefficient = 0.94, 95% confidence interval 0.88 – 0.98).[37] The KOOS is a 42-item self-administered questionnaire that includes five dimensions: pain, disease-related symptoms, activities of daily living function, sport and recreation function, and knee-related quality of life measured using a Likert scale (0–4 scale). For the current study, the KOOS was administered without the sport and related function dimension, so the KOOS contained 37 questions. Scores within a dimension or subscale are summed and scaled with zero corresponding to severe knee problems and 100 corresponding to no knee problems. Recently, the reliability and validity of the KOOS was examined in 105 patients with knee osteoarthritis after TKA surgery.[38] The test-retest reliability was adequate with intra-class correlation coefficients at least 0.75 for each of the KOOS subscales. When compared with the SF-36,[39] a widely used measure of general health status, expected and acceptable correlations were reported (e.g., KOOS pain and SF-36 bodily pain correlation coefficient = 0.62). The KOOS also includes dimensions that are not measured by the Western Ontario and McMaster Universities Osteoarthritis Index (WOMAC), including knee-related quality of life, which had high content validity with TKA candidates. Knee ROM was measured daily during the inpatient stay using a goniometer and standard procedure with the patient in sitting.[40]

The inpatient medical records were abstracted by two of the authors (JW and JC) for age, diagnosis, clinical status, date of admission (surgery), date of discharge, discharge disposition, and medical complications. Additionally, complications over the 6-month follow-up period were abstracted from outpatient medical records by the same two authors.

### Analysis

The purpose of this study was descriptive in nature, so analysis consisted of means and standard deviations for interval data, medians and inter-quartile ranges for ordinal data, and percentages for nominal data. Of the 74 patients who received a TKA, pre-operative data were available for 63 patients. Therefore, a sensitivity analysis was completed to determine if those who had missing pre-operative data had different characteristics than those who completed the preoperative data collection. SAS Version 9.13 was used for analysis (SAS Institute, Inc. Version 9.13, Cary, NC).

## Results

The patients were 71.4 ± 8.7 years old on average, and the majority (62%) was female. 45% were categorized as obese (BMI > 30 kg/m^2^), 38% were categorized as overweight (BMI 25–29.9 kg/m^2^) and 17% were categorized as normal weight (BMI 18–25 kg/m^2^).

**Figure 1:**
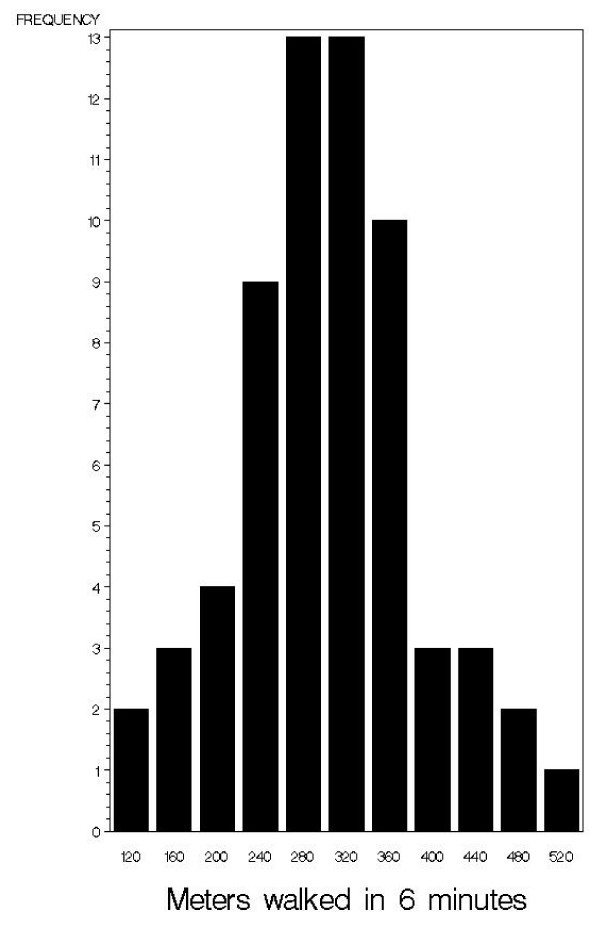
Six minute walk distance (m) for JRP patients who were seen preoperatively (n = 63).

Figure 1 shows the distribution of 6MWT distance in meters for the patients in the cohort who had a pre-operative visit. The distance walked ranged from 110 to 509 meters. To better understand the degree of functional limitation in this cohort of patients relative to the average population, a reference equation was applied to determine the predicted distance walked in six minutes adjusted for age, height, and weight.[41] Using this prediction equation, the patients walked distances on average 68% (± 16%) of age, height, and weight-matched peers.

The medians and inter-quartile ranges of the four dimensions of the KOOS for the 63 patients who were seen pre-operatively are presented in Table 2. The patients reported the most disability (lowest scores) in the quality of life dimension and the least disability (highest scores) in the disease-related symptoms dimension.

**Table 2:** Knee injury and osteoarthritis outcome score (KOOS) for JRP patients who were seen pre-operatively (n = 63)

**Dimension**	**Median (IQR)**
Disease related symptoms	57.1 (42.9 – 67.9)
Pain	44.5 (36.1 – 58.3)
Activities of daily living	52.9 (42.6 – 67.6)
Quality of life	25 (18.8 – 37.5)

IQR = interquartile range

The length of the inpatient hospital stay was 2 days for most (53%) of the patients, with 39% and 7% discharged in three and four days, respectively. Three patients (4%) were discharged to a skilled nursing facility. These three patients required more intensive rehabilitation than could be offered in a home or outpatient environment. The remaining patients were discharged home. Of those 71 discharged home, 68% received further rehabilitation in an outpatient setting compared with 32% who received home physical therapy for a period of time before attending outpatient physical therapy.

All patients who were discharged home achieved medical criteria for hospital discharge and met PT goals. For discharge home, PT goals included independence (with assistive device and/or caregiver if necessary) with transfers, ambulation greater than 150 feet, and a home-exercise program. For patients with stairs in their home environment, PT goals also included independence with an assistive device and/or caregiver. 88% of all patients achieved the knee flexion ROM goal of 90° (mean ROM 91.7 ± 5.4 °). 46% of all patients achieved the extension ROM goal of 0° (mean ROM 2.4 ± 2.6°).

Two of the 74 patients (3%) had medical complications during the inpatient hospital stay. One had transient arrhythmia and one had respiratory distress. These 2 patients were treated and the issues resolved prior to discharge from the hospital. During 6 months of post-discharge follow-up, there were 9 patients with complications requiring further care. One patient was readmitted on POD #3 with a diagnosis of dehydration. Intravenous fluids were administered and the patient was discharged without further incident. Three patients required oral antibiotics for the treatment of cellulitis. Two patients fell during this follow-up period. One fall occurred on POD #4 (2 days after discharge), and resulted in back pain that resolved with conservative treatment. The other fall occurred approximately 5 weeks after surgery, and the patient sustained a quadriceps tendon rupture. After surgical intervention and rehabilitation, a satisfactory outcome was reported. Three patients required knee-joint manipulations and additional physical therapy approximately 6–7 weeks post-surgery in order to increase flexion ROM.

A sensitivity analysis was completed comparing the 63 patients with pre-operative data with those who did not have pre-operative data collected. The results showed no statistical differences (p > 0.05 for all) for age, sex, hospital length of stay, or discharge disposition (data not shown).

## Discussion

The purpose of this descriptive study was to describe a wellness-based, comprehensive program (the JRP) for patients undergoing TKA and to report post-surgical outcomes of 74 consecutive inpatients. The program was developed based on current evidence and expert opinion and was designed to minimize lengths of stay and post-operative complications. The JRP consisted of pre-operative education, peri- and post-operative pain management, clinical pathways, and aggressive physical therapy. The majority of the patients in the JRP met the PT mobility goals (Table 1) to allow a safe discharge home. The JRP also resulted in short lengths of stay with few post-operative complications relative to published literature.[42]

The literature relating to pre-operative education prior to TKA is equivocal. A 2003 NIH Consensus Statement stated that pre-operative education is related to favorable outcomes.[12] Similarly, Coudeyre et al. concluded that a pre-operative program involving multidisciplinary education, exercise training, and discharge planning contributed to reduced hospital lengths of stay and improved functional status.[13] However, a Cochrane review of randomized controlled trials found little evidence to support the use of pre-operative education to improve pain, functioning, and length of hospital stay.[14] This review concluded that anxiety was the only variable that was consistently and positively impacted by pre-operative education.[14] The purpose of pre-operative education in our program was to lessen patients' anxiety by making them aware of post-operative rehabilitation and involving them in goal setting and discharge planning.

The current study administered a medication regimen designed to minimize post-operative pain, opiod use, and inflammation to encourage early and aggressive mobility. The pre-operative medication regimen included a long-acting analgesia and medication to reduce inflammation, anxiety, and nausea and vomiting. Intra-operative analgesia included a short acting spinal anesthesia. Regional anesthesia has been shown to result in less intra-operative blood loss, lower incidence of DVT, and better post-operative pain management compared with general anesthesia in patients undergoing hip surgery.[32] The results from the current study showed no incidence of DVT and short lengths of stay with discharge home, suggesting that pain was not a limiting factor in rehabilitation.

Prior to the surgical closing of the TKA, an injection of analgesics and anti-inflammatory medication was administered into the intra-articular space. This injection has been shown to improve pain control after joint arthroplasty. [15-17] In a trial of 64 patients admitted for TKA, 32 patients were randomized to receive a multimodal intra-operative injection and 32 patients comprised the control group.[15] Compared with the control group, those patients who received the injection used significantly less PCA in the immediate post-operative period (p < 0.01 at 6 hours, p = 0.016 at 12 hours, and p < 0.001 at 24 hours). The patients who received the injection also had higher patient satisfaction scores (p = 0.013) and lower scores for pain (p = 0.007) 4 hours after surgery compared with the control group. There were no significant between-group differences in the average length of hospital stay or incidence of complications. Although pain in the immediate post-operative period was not formally measured in the current study, the JRP protocol including the initiation of post-operative rehabilitation within 2–4 hours of surgery supports the hypothesis that the intra-articular injection provided good pain relief.

There is consistent support in the literature for early and aggressive physical therapy, or mobilization, following TKA.[10,21-24] A study of 50 patients reported a shorter length of stay for the experimental group who received early rehabilitation compared to the control group (3.6 ± 1.0 vs. 6.6 ± 2.6 days).[21] Interestingly, the working definitions of "early" and "aggressive" have changed over the years. Articles written several years ago defined early rehabilitation in "units" of days [10,22] and activity was often focused on chair sitting.[22] In contrast, recent work refers to early rehabilitation in terms of hours and activity in terms of active lower extremity exercise and ambulation.[21] Consistent with more recent literature,[10,21-25] the goal of our JRP program was to have patients ambulate 2–4 hours post-surgically. Although we cannot say definitively, we hypothesize that this early rehabilitation contributed to the attainment of JRP goals (Table 1) and lengths of stay that were short compared with published literature.[42]

Based on the reported results, the JRP program was considered successful. 71 out of the 74 patients were discharged home and were functionally independent or were safe with minimal assistance from a caregiver. At the time of discharge, knee ROM averaged 2.4° (± 2.6°) to 91.7° (± 5.4°). The average length of stay for patients in the JRP was 2.5 days. The authors see this result as optimal based on comparison with a recent study using the U.S. National Hospital Discharge Survey that reported an average length of stay for primary TKA of 5.3 days.[42] While a longer stay could result in higher costs to the hospital that are not likely to be reimbursed, a shorter stay may be associated with the failure to detect developing complications such as wound infection or cardiovascular conditions.[43] Despite the short lengths of stay, in-hospital complications occurred in fewer than 3% of the patients, which is lower than published data.[42] Post-discharge complications were reported in 12% of the patients during 6 months of follow-up.

The primary strength of this paper was that it included a clinical population of consecutive inpatients admitted for TKA to a regional medical center. All measurements and interventions were carried out by practicing clinicians as part of standard clinical care. Additionally, the characteristics of the patients reflect the demographics of TKA candidates.[3,42] The patients in the current study reported slightly higher pre-operative KOOS scores for each subscale compared with 105 TKA candidates,[38] but reported similar scores when compared with 47 people with severe osteoarthritis.[44] This study has some limitations. The design of this study was descriptive and therefore had no control group. This study compared results to published literature. It is important to understand and report characteristics of patients and thoroughly describe clinical programs as the basis for future clinical reference and/or research. The purpose of this study was to report outcomes of a comprehensive JRP; therefore this study was unable to evaluate isolated components of the program.

## Conclusion

In conclusion, we have shown that a comprehensive TKA program, consisting of pre-operative education, peri-operative pain management, and early and aggressive PT was associated with short lengths of stay, discharge home with outpatient physical therapy follow-up, and minimal inpatient and 6-month follow-up complications. This JRP may represent an efficient, effective and safe protocol for providing care after a TKA.

## Competing interests

The authors declare that they have no competing interests.

## Authors' contributions

JC conceived of the study, participated in data collection, provided patient care, and helped to draft the manuscript. MW performed the statistical analysis and helped to draft the manuscript. KG participated in the design of the study and helped to draft the manuscript. PP participated in the design of the study, and participated in data collection. JW helped with the design of the study, provided patient care, and helped to draft the manuscript. All authors read and approved the final manuscript.

## Pre-publication history

The pre-publication history for this paper can be accessed here:


